# Intracellular Complement Component 3 Attenuated Ischemia-Reperfusion Injury in the Isolated Buffer-Perfused Mouse Heart and Is Associated With Improved Metabolic Homeostasis

**DOI:** 10.3389/fimmu.2022.870811

**Published:** 2022-04-01

**Authors:** M-K. Torp, T. Ranheim, C. Schjalm, M. Hjorth, C.M. Heiestad, K. T. Dalen, P. H. Nilsson, T. E. Mollnes, S. E. Pischke, E. Lien, J. Vaage, A. Yndestad, K-O. Stensløkken

**Affiliations:** ^1^ Department of Molecular Medicine, Institute of Basic Medical Sciences, University of Oslo, Oslo, Norway; ^2^ Research Institute of Internal Medicine, Oslo University Hospital, Oslo, Norway; ^3^ Division of Surgery, Inflammatory Diseases and Transplantation, Institute of Clinical Medicine, University of Oslo, Oslo, Norway; ^4^ Department of Immunology, Institute of Clinical Medicine University of Oslo, Oslo, Norway; ^5^ Department of Nutrition, Institute of Basic Medical Sciences, University of Oslo, Oslo, Norway; ^6^ Linnaeus Centre for Biomaterials Chemistry, and the Department of Chemistry and Biomedicine, Linnaeus University, Kalmar, Sweden; ^7^ Stiftelsen Kristian Gerhard Jebsen (K.G. Jebsen) Inflammation Research Center (IRC), University of Oslo, Oslo, Norway; ^8^ Research Laboratory, Nordland Hospital, Bodø, and Faculty of Health Sciences, Stiftelsen Kristian Gerhard Jebsen (K.G. Jebsen) Thrombosis Research and Expertise Center (TREC), University of Tromsø, Tromsø, Norway; ^9^ Centre of Molecular Inflammation Research, Department of Cancer Research and Molecular Medicine, Norwegian University of Science and Technology, Trondheim, Norway; ^10^ Department of Research & Development, Division of Emergencies and Critical Care, Oslo University Hospital, Oslo, Norway; ^11^ Division of Infectious Diseases and Immunology, Program in Innate Immunity, Department of Medicine, UMass Medical School, Worchester, MA, United States; ^12^ Institute of Clinical Medicine, University of Oslo, Oslo, Norway

**Keywords:** ischemia-reperfusion injury, complement system, intracellular C3, metabolism, cardiology (basic/technical)

## Abstract

The innate immune system is rapidly activated during myocardial infarction and blockade of extracellular complement system reduces infarct size. Intracellular complement, however, appears to be closely linked to metabolic pathways and its role in ischemia-reperfusion injury is unknown and may be different from complement activation in the circulation. The purpose of the present study was to investigate the role of intracellular complement in isolated, retrogradely buffer-perfused hearts and cardiac cells from adult male wild type mice (WT) and from adult male mice with knockout of complement component 3 (C3KO). Main findings: (i) Intracellular C3 protein was expressed in isolated cardiomyocytes and in whole hearts, (ii) after ischemia-reperfusion injury, C3KO hearts had larger infarct size (32 ± 9% in C3KO vs. 22 ± 7% in WT; p=0.008) and impaired post-ischemic relaxation compared to WT hearts, (iii) C3KO cardiomyocytes had lower basal oxidative respiration compared to WT cardiomyocytes, (iv) blocking mTOR decreased Akt phosphorylation in WT, but not in C3KO cardiomyocytes, (v) after ischemia, WT hearts had higher levels of ATP, but lower levels of both reduced and oxidized nicotinamide adenine dinucleotide (NADH and NAD+, respectively) compared to C3KO hearts. Conclusion: intracellular C3 protected the heart against ischemia-reperfusion injury, possibly due to its role in metabolic pathways important for energy production and cell survival.

## Introduction

The innate immune system is rapidly activated during myocardial infarction (MI) and blockade of extracellular complement component 5 reduces infarct size in a porcine model (1, 2). mRNA of complement factor 3 (C3) is expressed in rabbit heart tissue and increases in hearts subjected to ischemia-reperfusion injury (3). To our knowledge, intracellular C3 protein expression has not been shown in the heart and the role of intracellular complement after ischemia-reperfusion of the heart is unknown. Since intracellular complement appears to be closely linked to metabolic pathways (4), its role in ischemia-reperfusion injury may not be the same as complement in the circulation.

MI is a main cause of morbidity and mortality worldwide, especially in developing countries (5, 6). Rapid reperfusion strategies remain the most important treatments of MI (7). Restoration of oxygen supply increases production of reactive oxygen species (ROS), which may facilitate opening of the mitochondrial permeability transition pore and ultimately cause cell necrosis (8). Release of endogenous damage associated molecular patterns (DAMPs) after myocardial necrosis activates the innate immune system. This includes the complement system, as demonstrated in clinical and *in vivo* experimental models of MI (9–11). Three different pathways can activate the complement system: the alternative pathway, the classical pathway, or the lectin pathway (12–14).

The most abundant protein within the complement system is C3, a protein composed of an α- and a β-chain (15). Upon pathogen invasion or tissue damage, extracellular C3 is either activated spontaneously or cleaved by one of the two C3-convertases (C4b2a or C3bBb) into C3a and C3b (14). C3a can activate the C3a receptor (C3aR) on cells inducing either pro- or anti-inflammatory responses (13, 16, 17). Certain complement proteins, including C3, also exist intracellularly in T lymphocytes, serving as a defense system against intracellular pathogen invasion (18, 19). Furthermore, intracellular C3 is closely linked to vital metabolic pathways in T cells and activation of intracellular C3aR in lysosomes by C3a has been suggested to signal *via* the mammalian target of rapamycin (mTOR) (4, 20, 21).

Our question is: what is the role of intracellular complement in cardiac ischemia-reperfusion injury when studied in isolated, buffer-perfused hearts from C3 knockout mice?

## Materials and Methods

Reagents were purchased from Sigma-Aldrich (St. Louis, MO) unless otherwise stated.

### Human Serum

A pool of normal human serum was prepared from whole blood obtained from six healthy volunteers. Blood sampling was approved by the Regional Ethics Committee of the South-Eastern Norway Regional Health Authority. The donors gave informed written consent.

### Animals

Male C3KO (C3^-/-^) and WT (C3^+/+^) littermate mice of C57BL/6J background were bred at Radiumhospitalet, Oslo, Norway. Mice with the C3KO allele were originally obtained from The Jackson laboratory (Bar Harbor, ME). The animals were kept in an environment of 12:12 hours light:dark cycle, at a temperature of 23°C, and 55-60% humidity. Water and chow were available *ad libitum*. All animal experiments were performed in accordance with the Norwegian Food Safety Authority (FOTS ID 12108) and the guidelines from Directive 2010/63/EU of the European Parliament on the protection of animals used for scientific purposes. For all animal experiments, mice (age; 8.2 ± 0.9 weeks, weight; 25.8 ± 2.2 grams) were anesthetized with intraperitoneal injection of sodium pentobarbital (50 mg/kg) and heparin (500 IU, Leo Pharma A/S, Denmark) before they were euthanized by cervical dislocation and heart excision.

### Langendorff Heart Perfusion

The method has been described previously (22). Briefly, hearts of either C3KO or WT mice were harvested and quickly cannulated through the aorta, facilitating retrograde perfusion at constant pressure (70 mmHg) with Krebs-Henseleit buffer (in mM: NaCl 118.5, NAHCO_3_ 25.0, KCl 4.7, KH_2_PO_4_ 1.2, MgSO_4_/7H_2_O 1.2, glucose/1H_2_O, 11.1, CaCl_2_ 2.4). Order of the genotypes was randomly selected by drawing. A handmade, fluid-filled, plastic balloon was inserted into the left ventricle (LV) to monitor ventricular pressures with Powerlab and Labchart (ADInstruments Ltd, Oxford, UK). Left ventricular end-diastolic pressure (LVEDP) was adjusted to 5-10 mmHg during stabilization (22). Hearts that exceeded the following criteria were excluded from the study: Aortic cannulation time >3 minutes, coronary flow <1 and >4 mL/min, LV systolic pressure (LVSP) <60 mmHg, heart rate <220 beats per minute (bpm). After 20 minutes of stabilization, 35 minutes of global ischemia was followed by 60 minutes of reperfusion. At the end of the experiment, hearts were sectioned into four 1 mm slices, which were stained with 1% triphenyltetrazolium chloride (TTC) for blinded assessment of infarct size (Adobe Photoshop version 13.0). One 2 mm slice was fixed in 4% formalin solution for subsequent immunohistochemistry. After 10 minutes of stabilization and at 1, 3, 5, 10, 15, 20, 30, 40, 50, and 60 minutes of reperfusion, coronary perfusate was collected for one minute and analyzed for lactate dehydrogenase (LDH) (Cytotoxicity Detection Kit, Roche, Penzberg, Germany).

### Cardiomyocyte Isolation

Primary adult cardiomyocytes were isolated from hearts excised as described above and isolated according to O’Connell et al. (23). Hearts were initially perfused with perfusion buffer (in mM: NaCl 120.4, KCl 14.7, KH_2_PO_4_ 0.6, Na_2_PO_4_ 0.6, MgSO_4_ 1.2, Na-HEPES liquid 10.0, glucose 5.5, NAHCO_3_ 4.6, taurine 30.0, BDM (2,3-butanedione monoxime) 10), and subsequently digested with 1.3 mg/mL collagenase type 2 (#4177, batch 45D15719, activity 355 U/mg, Worthington Biochemical, Lakewood, NJ). The collagenase was inhibited with HyClone^®^ bovine calf serum (FBS, #SH30073.03, GE Healthcare Life Sciences, Marlborough, MA) diluted in perfusion buffer. Cardiomyocytes were separated by gently pipetting and centrifugation at 20 × *g* to separate cardiomyocytes from non-cardiomyocytes. The cardiomyocyte suspension was purified by repeated centrifugations at 20 × *g* with increasing concentration of Ca^2+^, resuspended in plating medium [MEM with Hanks BSS (#M5775)] supplemented with 10% FBS, 100 U/mL penicillin-streptomycin, 2 mM L-glutamine, and 10 mM BDM), and plated on laminin-coated plates (1 µg/cm^2^, #354232, Corning, NY). Plating medium was replaced by short term medium (MEM with Hanks BSS supplemented 100 U/mL penicillin-streptomycin, 2 mM L-glutamine, 0.1% BSA (low endotoxin, fatty acid free), and 1 mM BDM). Cardiomyocytes were incubated in 2% CO_2_ at 37°C.

### High-Resolution Respirometry

#### Cardiac Mitochondria Isolation

Cardiac mitochondria were isolated from C3KO and WT hearts perfused with ice cold 1X PBS. The hearts were homogenized at maximal speed (IKA^®^ ULTRA-TURRAX^®^ T-8 tissue homogenizer) in isolation buffer (in mM: sucrose 250, Na_2_ –EDTA 0.5, and Tris 10, 3 µg/mL proteinase type XXIV, adjusted to pH 7.4 at 4°C) for seven seconds. The homogenate was incubated on ice for 10 minutes, before isolation buffer with 0.1% BSA (low endotoxin, fatty acid free) was introduced. Initially, homogenates were centrifuged at low speed (600 × *g*) to separate the mitochondria. The mitochondria isolate was purified by three times high speed centrifugation (9800 × *g*, 9600 × *g*, and 9200 × *g*). The mitochondria were resuspended in MSHE buffer (in mM: D-mannitol 210, D-sucrose 70, HEPES 10, EGTA 1, EDTA 1, adjusted to pH 7.4).

#### Permeabilized Primary Cardiomyocytes

Cardiomyocytes were isolated as described above, except that cells were resuspended in ice cold MitoMed solution (in mM: sucrose 110, lactonate 60 mM, K_2_EGTA 0.5, MgCl_2_ 3, dithiothreitol 0.5, taurine 20, KH_2_PO_4_ 3, HEPES 20, adjusted to pH 7.1) (24). Cardiomyocytes were permeabilized with 20 µg/ml saponin for 10 minutes on ice allowing intracellular substrate entry, and subsequently washed three times with ice-cold MitoMed solution.

#### Oxygraph-2k High-Resolution Respirometry Experiment

Isolated mitochondria or permeabilized cardiomyocytes were added in an Oxygraph-2k (Oroboros Instruments, Innsbruck, Austria) together with MiR05 buffer (in mM: sucrose 110, K-lactobionate 60 mM, EGTA 0.5, MgCl_2_ 3, taurine 20, KH_2_PO_4_ 10, K-HEPES 20, BSA 1 mg/mL, adjusted to pH 7.1), 10 mM glutamate, 5 mM pyruvate, and 2 µM malate, and kept at 37°C, in order to measure basal respiration. After stabilization, the following was added; 1 mM ADP (complex I activity), 0.1 nM rotenone (inhibits complex I), 10 mM succinate (complex II activity), 2.5 µM antimycin A (inhibits complex III), and 2 mM ascorbate and 0.5 mM TMPD (complex IV activity). Anoxia/reoxygenation was obtained by waiting for the oxygen concentration to reach zero and leave the permeabilized cells for 35 minutes without oxygen, before oxygen was re-introduced for 10 minutes. Substrates were then added as described above. All oxygen consumption measurements were normalized to protein concentration.

### ^14^C-glucose Uptake in Primary Cardiomyocytes

Cardiomyocytes cultured in 12-well plates were incubated in glucose-free medium and treated with 100 nM insulin (Humalog, Eli Lilly and Company, Indianapolis, IN) for 30 minutes. To measure glucose uptake, 0.2 mM 2-deoxyglucose mixed with 0.077 µCi/ml [14C]2-deoxyglucose (Perkin-Elmer, Waltham, MA) was added to the cell culture medium for additional 30 minutes (specific activity 385 µCi/mmol). The cells were washed three times in PBS, harvested in 0.1 M NaOH and the lysed cells counted by conventional liquid scintillation with OptiPhase SuperMix scintillation fluid (Perkin-Elmer). Glucose uptake was normalized to the cell lysate protein content.

### Metabolomic Analysis of WT and C3KO Mouse Heart Tissue

Hearts from WT and C3KO were excised and briefly perfused with 5% mannitol solution before the hearts were snap frozen in liquid nitrogen. Heart tissues were crushed in liquid nitrogen and each sample was weighed (48.76 ± 0.49 mg) for untargeted metabolomic analysis. The metabolome of six WT and six C3KO hearts were analyzed by Human Metabolome Technologies Inc., Tsuruoka, Japan, according to in-house protocols.

### Metabolite Measurements

Hearts from WT and C3KO mice were excised and retrogradely perfused as described in the Langendorff heart perfusion section. After 20 minutes stabilization, hearts were exposed to 35 minutes ischemia followed by 1 minute reperfusion. Hearts were immediately frozen at 1minute reperfusion and levels of nicotinamide adenine dinucleotide (NAD) and ATP were detected by luminescence assays and quantified according to standard curves. NAD^+^ and NADH were analyzed with NAD/NADH-Glo™ Assay (Promega, Madison, WI) and the ATP levels were analyzed with ATPlite (Perkin-Elmer), both according to the manufacturers’ protocols.

### TUNEL Assay

Mouse hearts were sectioned into 2 mm thick slices, fixed in 4% formalin solution for 24 hours, and subsequently stored in 70% ethanol at 4°C. The hearts were embedded in paraffin and cut into 3 µm thick sections. Sections were deparaffinized with xylene and rehydrated in graded ethanol washes (100%, 95%, 85%, 70%, and 50%). Sections were permeabilized with 20 µg/mL Proteinase K for 15 minutes at room temperature. Apoptosis was analyzed with DeadEnd™ Fluorometric TUNEL System (Promega, Madison, WI) and was used according to protocol. The TUNEL stained sections were mounted in ProLong Gold Antifade Reagent with DAPI (Thermo Fisher Scientific, Waltham, MA). Slides were analyzed with a Zeiss high-throughput microscope (Carl Zeiss AG, Oberkochen, Germany) at 20× magnification. Twenty-five images were automatically captured (approximately 6500 nuclei on average), and number of TUNEL stained nuclei and the total number of nuclei were objectively analyzed and counted with Cell profiler™ cell image analysis software (25).

### Western Blotting

Tissue and cells were lysed in 1X RIPA lysis and extraction buffer (Thermo Fisher Scientific), sonicated, and heated in 5X sample loading buffer (*non-reducing*: 60 mM Tris-HCl, pH 6.8, 25% glycerol, 2% SDS, and 0.1% bromophenol blue; *reducing*: 60 mM Tris-HCl, pH 6.8, 25% glycerol, 2% SDS, and 0.1% bromophenol blue, and 5.7% β-mercaptoethanol). Protein extracts were separated on a 4-20% Criterion Pre-cast gel (BioRad, Hercules, CA) at 200 V in Tris/glycine/SDS buffer and transferred to a nitrocellulose membrane (BioRad) at 100 V in ice-cold Tris/glycine/methanol buffer. Total loaded proteins were stained with 0.1% Ponceau solution (Merck-Millipore, Burlington, MA). Membranes were blocked in 5% non-fat dry milk (BioRad) for 1 hour in room temperature, before primary antibody labelling at 4°C overnight. Primary antibodies (anti-C3 (1:1000, #ab200999, Abcam, Cambridge, United Kingdom), anti-Akt (1:1000, #9272, Cell Signaling, Danvers, MA), anti-Phospho-Akt (Ser473; 1:000, #9271, Cell Signaling), anti-mTOR (1:1000, #2972, Cell Signaling), and anti-phospho-mTOR (Ser2448; 1:1000, #2971, Cell Signaling)) were detected with HRP-conjugated secondary antibodies for 1 hour in room temperature. For visualization, membranes were incubated with SuperSignal™ West Dura Extended Duration Substrate (Thermo Fisher Scientific), and developed with ChemiDoc Touch Imaging System (BioRad). Band intensity was analyzed with Image Lab Software (BioRad).

### ELISA

ELISA kit were purchased from R&D systems (R&D Systems Inc. Minneapolis, MN) and the protocol was conducted according to the manufacturer. In short, capture antibodies were diluted in PBS and added to high-binding 96-well plates 1 day in advance of the experiment. Plates were blocked with reagent diluent (1% BSA in PBS) and washed with washing buffer (0.05% Tween 20 in PBS). Substrate solution was purchased from Life Technologies (TMB Single solution, Thermo Fisher Scientific Inc.), and H_2_SO_4_ was used as stop solution. Absorbance was measured at 450 and 570 nm with BioTek PowerWave XS (BioTek Instruments Inc.).

### Statistical Analysis

The statistical analyses were performed with Graphpad Prism version 8 Software (GraphPad Software, Inc., San Diego, CA). Unless otherwise stated, data sets were analyzed with repeated measures two-way ANOVA and Bonferroni’s multiple comparison test, or two-tailed t-tests. The statistical analyses are presented as scatterplots with median or mean ± SEM. p<0.05 was considered statistically significant. Data sets were tested for normality, and proper statistical tests were chosen accordingly. The experimental protocol in the high respirometer experiments gave day-to-day variations, thus the data are paired according to day and substrates within genotype. Differences in mean for each complex activity assessment are then analyzed with Bonferroni’s multiple comparison test.

## Results

### Intracellular C3 Protein Expression in the Heart

**Figure 1:**
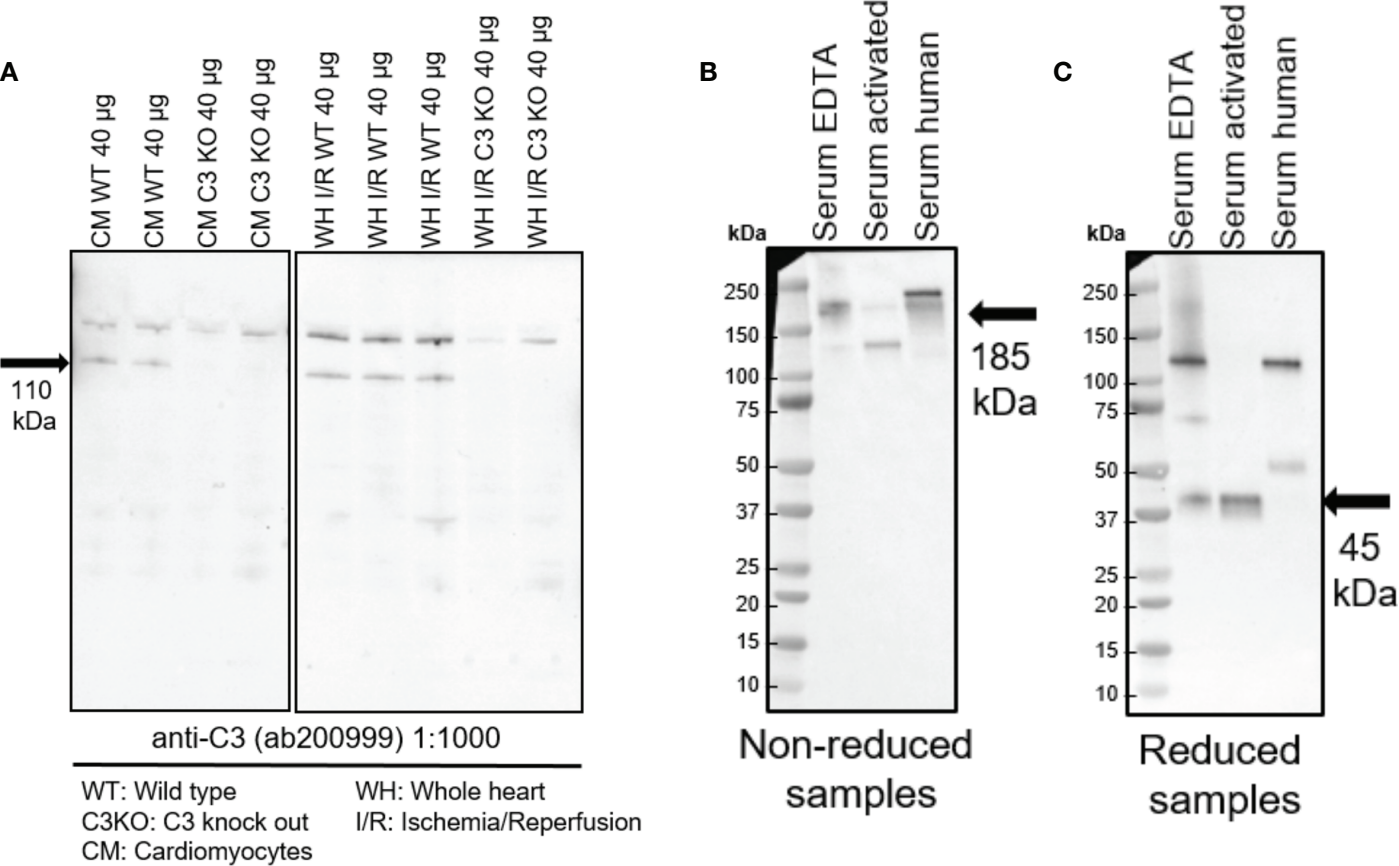
Western blots showing expression of intracellular C3 in the heart. **(A)** Equal amounts of cardiomyocytes and heart tissue were loaded in a 4-20% gradient gel. A band at 110 kDa band was observed in cardiomyocytes (CM) and whole heart tissue lysate from ischemia-reperfused hearts (WH I/R) from wild type (WT) mice, which was not present in the complement component 3 knock out (C3KO) samples. Serum and activated serum from WT mice and human serum were tested at both reduced **(B)** and non-reduced conditions **(C)**. A band at 185 kDa (non-reduced) and 110 kDa (reduced) corresponding to intact α-chain of C3 was observed in WT serum and human serum **(B)**. Cleaved C3 α-chain (45 kDa) was observed in the reduced samples **(C)**.

Equal amounts of proteins of cardiomyocytes and heart tissue lysate from hearts exposed to ischemia-reperfusion injury were analyzed by western blotting and showed a band at 110 kDa (**Figure 1A**). This ubiquitously expressed band, corresponding to the size of intact C3 α chain (26), was absent in heart tissue and cardiac cells from C3KO mice (**Figure 1A**). Antibody specificity was confirmed in serum and activated serum from WT mice and human at both reduced and non-reduced conditions (**Figures 1B, C**). Bands at 185 kDa (non-reduced) and 110 kDa (reduced) were observed, corresponding to intact α chain in WT serum and human serum. Moreover, the 45-kDa C-terminal part of the C3 α chain, generated after complement factor I-cleavage (27), was observed in the reduced samples (**Figure 1C**).

### Intracellular C3 Attenuated Myocardial Ischemia-Reperfusion Injury

**Figure 2:**
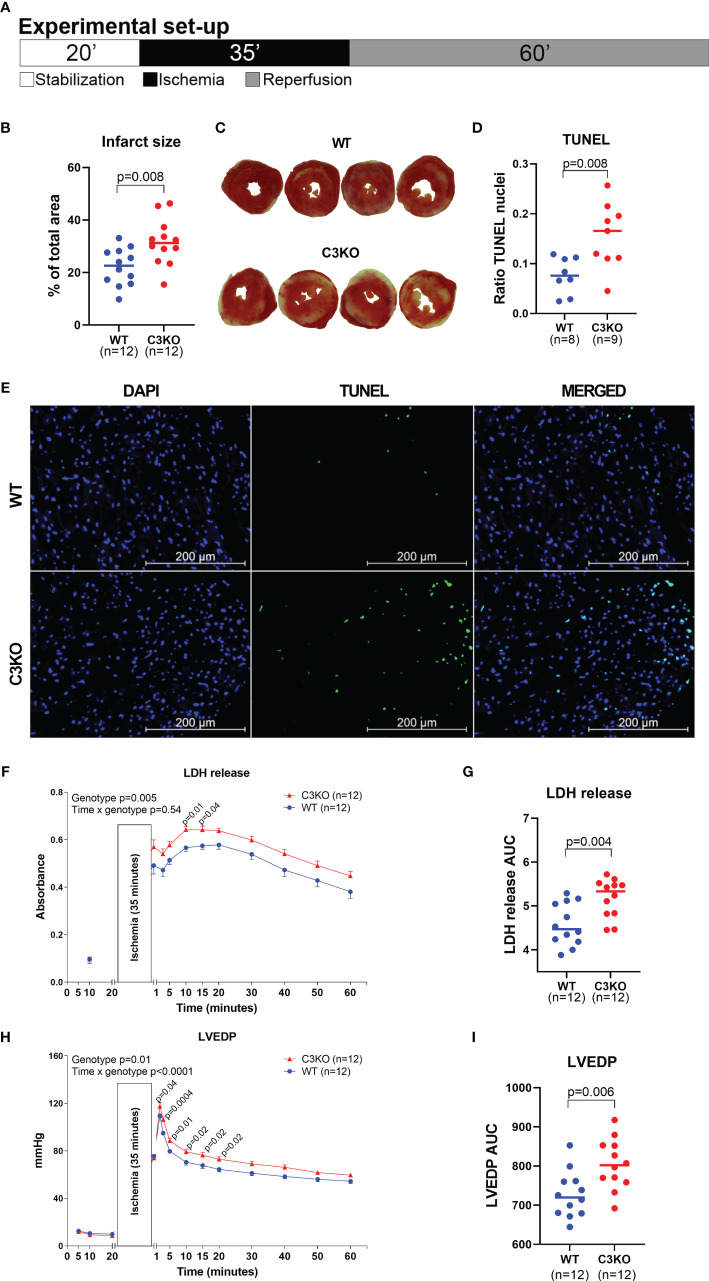
Langendorff heart perfusion. Retrograde buffer perfusion of isolated wild type (WT) and complement component 3 knock out (C3KO) hearts exposed to ischemia-reperfusion. Hearts were exposed to 20 minutes stabilization, 35 minutes ischemia and 60 minutes of reperfusion as indicated in panel **(A)**. **(B)** Infarct size measurements after reperfusion with images of hearts representing the mean infarct size value in each genotype **(C)**. **(D)** Ratio of TUNEL stained nuclei over total number of nuclei in heart sections after ischemia-reperfusion, and **(E)** representative images of TUNEL and DAPI stained heart sections (20× magnification, Scale bar: 200 µm). **(F)** Lactate dehydrogenase (LDH) release, corresponding to tissue damage, measured in coronary perfusate, also presented as area under curve **(G)**. **(H)** Left ventricular end-diastolic pressure (LVEDP) throughout the ischemia-reperfusion experiment, also as area under curve **(I)**. Data sets are displayed as scatterplots with median or mean ± SEM.

C3 deficiency in isolated hearts, *i.e.* without circulating blood or serum proteins, exacerbated myocardial damage after ischemia-reperfusion (**Figure 2**). Following ischemia-reperfusion, infarct size was 32 ± 9% in C3KO hearts compared to 22 ± 7% in WT hearts (**Figures 2A–C**). C3KO hearts had two-fold more TUNEL positive apoptotic cells compared to WT hearts after ischemia-reperfusion (**Figures 2D, E**). LDH release into coronary perfusate from C3KO hearts was 13% higher than from WT hearts (**Figures 2F, G**). Post-ischemic relaxation was moderately impaired in C3KO hearts compared to WT hearts, demonstrated by increased left-ventricular end-diastolic pressure (LVEDP; **Figures 2H, I**) and heart rate (**Supplementary Figure S1A**), measured isovolumetrically by a balloon in the left ventricle. Other parameters such as rate pressure product (RPP), coronary flow, and LV developing pressure (LVdevP) did not differ between groups (**Supplementary Figures S1A, B–D**). Baseline performance at the end of 20 minutes stabilization is presented in **Table 1**. Moreover, IL-6 release into the coronary perfusate did not show any differences between WT and C3KO hearts (**Supplementary Figure S5**).

**Table 1:** Baseline performance at end of 20 minutes stabilization.

	WT (mean ± SD)	C3KO (mean ± SD)	*p-value*
Number of animals	12	12	–
LVEDP (mmHg)	9.88 ± 4.3	8.56 ± 2.4	0.358
Heart rate (bpm)	340.9 ± 42.3	367.1 ± 71.7	0.287
LVdevP (mmHg)	117.5 ± 7.8	119.4 ± 7.7	0.559
RPP	40,098 + 5845	43,531 + 8218	0.251
Coronary flow (ml/minute)	2.29 ± 0.5	2.05 ± 0.3	0.205
Body weight (grams)	25.1 ± 1.9	26.5 ± 2.4	0.073
Mouse age (weeks)	8.3 ± 1.0	8.2 ± 0.8	0.962

LVEDP, Left-ventricular end-diastolic pressure; bpm, beats per minute; LVdevP, Left­ventricular developing pressure; RPP, rate pressure product. t-tests were used to calculate the p-values.

### Intracellular C3 Regulates Mitochondrial Respiration in Cytosol

**Figure 3:**
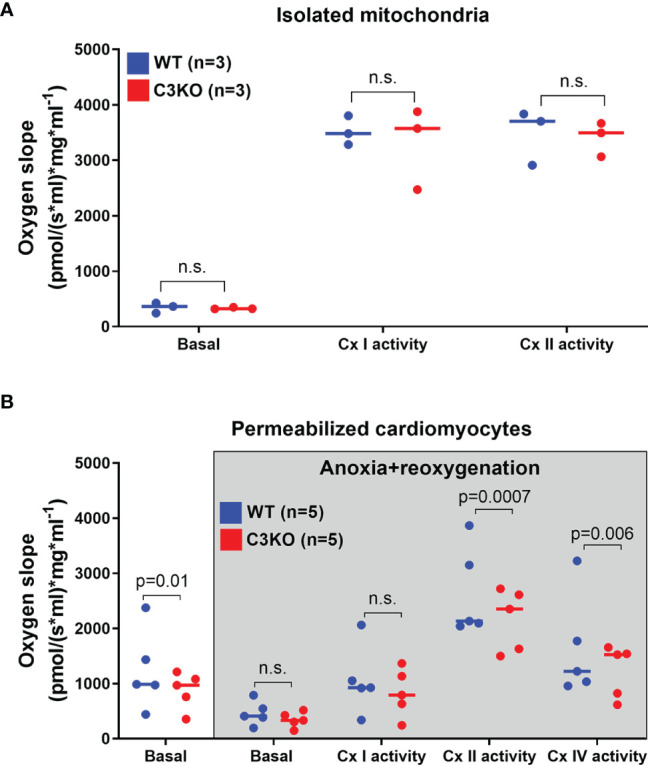
Mitochondrial respiration in WT and C3KO cardiomyocytes. Mitochondrial respiration (oxygen slope) was measured in **(A)** isolated mitochondria from hearts of wild type (WT) and mice with knock out of complement component 3 (C3KO) exposed to different substrates and mitochondrial complex inhibitors with high-resolution respirometry (Oroboros Oxygraph-2k). **(B)** Oxygen consumption was also measured in permeabilized cardiomyocytes that underwent anoxia for 35 minutes, similar as Langendorff experiment (**Figure 2**), with subsequent 10 minutes of reoxygenation. Data sets are displayed as scatterplots with median. ns, not significant.

Mitochondrial respiration in isolated cardiac mitochondria from whole heart and permeabilized cardiomyocytes from WT and C3KO mice was assessed (**Figure 3**). Mitochondria from WT and C3KO did not show any differences in oxygen consumption (**Figure 3A**), neither for basal respiration nor activity of electron transport chain protein complex I and II, suggesting no direct interaction between C3 and mitochondrial function. However, basal oxygen consumption in permeabilized C3KO cardiomyocytes was lower than in WT cardiomyocytes (**Figure 3B**). To mimic ischemia-reperfusion in hearts, we exposed permeabilized cardiomyocytes from WT and C3KO to 35 minutes of anoxia followed by 10 minutes reoxygenation. After reoxygenation, the activity of mitochondrial electron transport chain protein complex II and IV were significantly reduced in permeabilized cardiomyocytes from C3KO compared to WT (**Figure 3B**), suggesting that intracellular C3 supports mitochondrial respiration in cardiomyocytes.

### Role of Intracellular C3 in mTOR Signaling and Glucose Uptake

**Figure 4:**
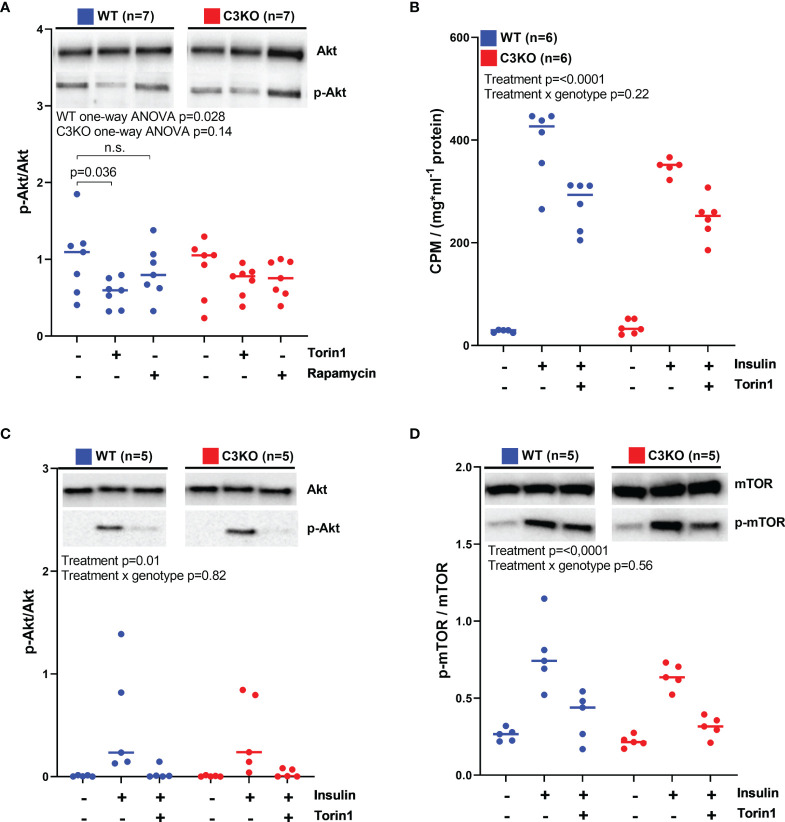
mTOR signaling and glucose uptake in WT and C3KO cardiomyocytes. **(A)** Activation of Akt was measured in cardiomyocytes from wild type (WT) and complement component 3 knock out (C3KO) that were treated with the mTOR inhibitors, 100 nM Torin1 or 50 nM rapamycin. Activation of Akt was quantified as a ratio between phosphorylated Akt (p-Akt) over total Akt. Data were analyzed with one-way ANOVA and Dunnett’s multiple comparison test for each genotype. **(B)** ^14^C-labelled glucose uptake was characterized in cardiomyocytes from WT and C3KO treated with 100 nM Torin1. **(C)** Akt and **(D)** mTOR activation were investigated in cardiomyocytes treated with 100 nM Torin1 with western blotting. Data sets are displayed as scatterplots with median. ns, not significant.

The mTOR signaling pathway was assessed, and in primary cardiomyocytes, no difference in Akt phosphorylation was found between WT and C3KO (**Figure 4A**). However, Akt phosphorylation was reduced by 50% in WT cardiomyocytes treated with the mTOR inhibitor Torin1 (**Figure 4A**). Whereas, Torin1 treatment had no effect in C3KO cardiomyocytes. Rapamycin did not influence Akt phosphorylation in neither genotypes, suggesting that C3 may have a function upstream of mTORC2, as rapamycin is mTORC1-specific and Torin1 inhibits mTORC1 and mTORC2 (28). Akt and mTORC2 are important mediators in the insulin-mediated glucose-uptake signaling cascade (29). However, glucose uptake in insulin-treated cardiomyocytes did not reach statistically differences between WT and C3KO (**Figure 4B**). Similarly, we found no genotype dependent differences in phosphorylation of neither Akt (**Figure 4C**) nor mTOR (**Figure 4D**) in insulin-treated cardiomyocytes. Treatment of cardiomyocytes from WT mice with extracellular human or mouse C3a did not influence Akt phosphorylation (**Supplementary Figure S2**).

### Intracellular C3 Influenced the Level of Nicotinamide Adenine Dinucleotide and ATP

**Figure 5:**
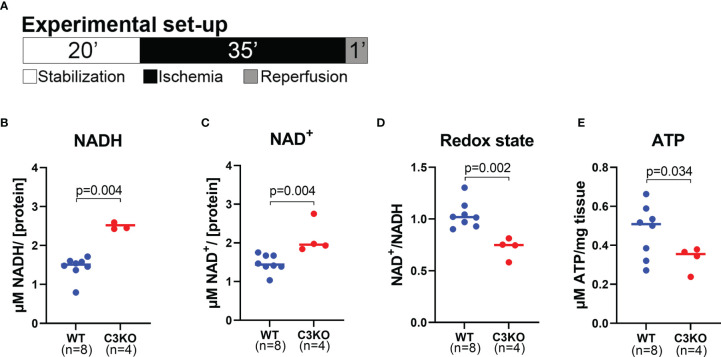
Metabolites in mouse WT and C3KO hearts. Isolated hearts from wild type (WT) and Complement component 3 knock out (C3KO) were exposed to 20 minutes stabilization, 35 minutes ischemia, and 1 minute reperfusion **(A)**, and selected metabolites were measured by luminescence assays. **(B)** NADH, **(C)** NAD+, **(D)** NAD^+^/NADH ratio, and **(E)** ATP were quantified in heart tissue and normalized to protein concentration **(B–D)** or mg tissue **(E)**, n=4-8. Panel B and C did not pass normality test and are analyzed with a non-parametric Mann-Whitney t-test. Data sets are displayed as scatterplots with median.

To investigate metabolite levels at a basal level, isolated hearts from WT and C3KO mice were briefly perfused for <30 seconds with 5% mannitol solution. The untargeted metabolomic analysis detected 237 metabolites with 16 metabolites significantly different between hearts from C3KO and WT (**Supplemental Figure S3**). C3KO hearts had reduced NAD (NADH) levels compared to WT hearts (**Supplemental Figure S3K**). NAD is central in numerous metabolic pathways, including glycolysis and oxidative phosphorylation, and once impaired causes increased tissue injury (30, 31). As the NADH levels were lower in C3KO hearts, we wanted to investigate the NAD state in the acute phase after ischemia. NAD was therefore investigated in isolated hearts from WT and C3KO exposed to 35 minutes of ischemia and one minute of reperfusion (**Figure 5A**). The levels of NADH (**Figure 5B**) and NAD^+^ (**Figure 5C**) were higher in C3KO hearts compared to WT hearts. Moreover, an impaired redox state was observed in the C3KO hearts compared to WT (**Figure 5D**). Relatable to the redox state, C3KO hearts had significantly lower ATP levels after ischemia compared to WT (**Figure 5E**). Release of lactate and succinate in the coronary perfusate were investigated, and although largely increased after ischemia, we found no genotype dependent differences (**Supplementary Figure S4**).

## Discussion

In this study, we provide evidence for the existence of intracellular C3 protein in cardiomyocytes and describe distinct functions of this protein. Intracellular C3 protected the heart against ischemia-reperfusion tissue injury and improved post-ischemic cardiac function. The use of isolated, buffer-perfused hearts without blood and plasma excluded a possible effect of plasma C3. The present findings place intracellular C3 central in metabolic pathways important for energy production and cell survival.

### Isolated, Perfused Hearts and Complement Component 3

In a glucose-rich, isolated heart perfusion set-up, C3KO hearts had larger infarct size after ischemia-reperfusion and more LDH release in the coronary perfusate compared to WT. Already after one minute of reperfusion, C3KO had higher LDH release compared to WT hearts. Hearts from C3KO mice had higher LVEDP during reperfusion, indicating increased stiffness of the left ventricle compared to WT hearts. All taken together, this indicates that intracellular C3 is involved in the acute processes of ischemia-reperfusion injury.

The complement system has been extensively studied as a potential therapeutic target after MI *in vivo*, where inhibition of extracellular C3 activation is protective in experimental models (32–34). However, studies in C3KO mice reported aggravated scar formation, less viable myocardium, and more apoptosis 28 days after coronary artery ligation *in vivo* compared to WT mice (35). It is not known if this effect is caused by lack of intracellular or extracellular C3. In the present study, C3KO hearts had more post-ischemic apoptosis. Blocking CTSL, responsible for intracellular cleavage of C3, increased apoptosis in T lymphocytes (4). CTSL is ubiquitously expressed in the heart, and CTSL-deficient mice had poorer survival rate and adverse post-MI remodeling (36). These findings support a role for intracellular C3 in upstream apoptotic signaling. Moreover, the role of C3 and C3aR in regulation of apoptosis are supported by C3aR KO mice showing aggravated liver apoptosis compared to WT mice after infection with the intracellular invading bacteria *Listeria monocytogenes* (37). Although intracellular complement activation is mainly investigated in T cells, intracellular C3 is expressed in different cell types, suggesting a general homeostatic role (38).

### Cardiomyocytes, Mitochondrial Respiration, mTOR, and Complement Component 3

C3KO cardiomyocytes had reduced oxidative phosphorylation after anoxia-reoxygenation compared to WT cardiomyocytes. However, isolated mitochondria had no genotype dependent differences, suggesting that C3 may have a metabolic role in the cytosol influencing mitochondrial oxidative phosphorylation. The major cellular challenge of ischemia-reperfusion is the lack of oxygen, extracellular increase of pH, and subsequently decreased ATP production with coherent detrimental consequences. After ischemia, ATP levels in C3KO hearts were reduced compared to WT hearts, most probably caused by lower mitochondrial respiration. A reduction in resting state oxygen consumption was observed in myocardial homogenate of CTSL deficient mice (39), supporting the importance of activating intracellular C3 in the heart for mitochondrial respiration. In our experiments, we studied mitochondrial respiration in cardiomyocytes in a high-resolution respirometer, containing buffer with good buffer capacity limiting pH variations, which does not directly mimic I/R in whole hearts.

Activation of C3aR by C3a has been suggested to signal through mTOR (20, 21). mTOR plays a pivotal role in metabolic activity and cell survival, and inhibition of C3 cleavage reduced mTOR activity in T cells (40, 41). It has been shown that treatment with siRNA against C3aR expression in T cells reduces mTOR activation and decreases cell survival. Treatment with extracellular C3a does not activate mTOR in these cells (4), similar to our finding where extracellular C3a treatment of cardiomyocytes had no effect on Akt phosphorylation. mTOR can be part of two different protein complexes, mTORC1 or mTORC2 protein complexes (29). mTORC2 is involved in cellular survival through direct phosphorylation and activation of Akt, which in turn facilitates insulin-mediated glucose uptake (42–45). When inhibiting mTOR in cardiomyocytes at a basal, non-treated level with rapamycin (inhibits mainly mTORC1) and Torin1 (inhibits both mTORC1 and mTORC2) (28), cardiomyocytes from WT had significantly lower Akt activation with Torin1 inhibition. This was not observed in C3KO cardiomyocytes, and may indicate a possible role of C3 in activation of mTORC2-Akt pathway. In our isolated heart and cardiomyocyte experiments, the only available energy-providing nutrient was glucose. mTORC2-Akt activation is responsible for translocation of the glucose transporters Glut4 to the plasma membrane and facilitate glucose uptake (29, 45). Consequently, C3KO hearts may have less intracellular glucose available before initiation of ischemia, which may cause a poorer post-ischemic outcome. However, we detected no significant differences in glucose uptake between C3KO and WT cardiomyocytes, neither at the basal level nor with insulin treatment. Furthermore, insulin initiated phosphorylation of both Akt and mTOR in cardiomyocytes, but intracellular C3 appeared uninvolved in this experiment. In our experiment, isolated cardiomyocytes are quiescent and the energy demand is very low compared to beating hearts (46). Previous studies have shown that insulin-stimulated glucose uptake and oxidation are lower in quiescent cardiomyocytes compared to beating hearts (47). Consequently, we cannot exclude that intracellular C3 may participate in mTOR signaling and glucose uptake in the energy-demanding beating heart.

### Metabolites and Complement Component 3

NAD plays a central and vital role in several metabolic pathways essential for cellular energy metabolism (31). The NAD^+^ and ATP levels are strictly controlled (48). A build-up of NADH levels is observed in respiration-impaired cells and are proven to be highly detrimental to the myocardium (49, 50). Moreover, imbalance in redox state, ratio between oxidized NAD (NAD^+^) and reduced NAD (NADH), results in cardiac dysfunction in a model for chronic diabetes (51). To our knowledge, no studies have reported the levels of different metabolites concerning intracellular C3. As discussed above, C3 is likely involved in the acute processes of ischemia-reperfusion injury. After exposure to ischemia and one minute of reperfusion, C3KO hearts had increased levels of NADH and NAD^+^ compared to WT hearts. Furthermore, redox state was balanced in WT hearts, but reduced in C3KO hearts. Consequently, intracellular C3 was important in cell metabolism and survival.

## Conclusions

The present findings identified a beneficial role of intracellular C3 in the oxygen-deprived myocardium. Intracellular C3 appears to be an important player in metabolic homeostasis during cardiac ischemia-reperfusion injury, which is in addition to the inflammatory effects associated with extracellular complement activation (2). Understanding the basic metabolic signaling and the exact role of intracellular C3 in the oxygen-deprived heart may improve treatment strategies of myocardial infarction and metabolic diseases.

## Data Availability Statement

The original contributions presented in the study are included in the article/**Supplementary Material**. Further inquiries can be directed to the corresponding author.

## Ethics Statement

The studies involving human participants were reviewed and approved by Regional Ethics Committee of the South-Eastern Norway Regional Health Authority. The patients/participants provided their written informed consent to participate in this study. The animal study was reviewed and approved by Norwegian Food Safety Authority (FOTS ID 12108).

## Author Contributions

M-KT have performed a major part of the experiments and analysis regarding the isolated heart perfusion. TR performed the TUNEL assay. M-KT and TR did the cardiomyocytes isolation and mitochondria respiration experiments. M-KT and CMH performed the western blotting. MH and KD performed the glucose uptake measurements. The mouse model was established by EL and CS was responsible for the mouse breeding. AY, K-OS, PN, TM, SP and JV contributed in development of hypotheses, data interpretation and manuscript writing. AY and K-OS supervised the study. M-KT wrote the initial manuscript. All authors have contributed to the final manuscript.

## Funding

This work was supported by the University of Oslo through the Molecular Life Science (MLS) program, Research Council of Norway Center of Excellence Funding Scheme project 223255/F50, and Nansenfondet.

## Conflict of Interest

The authors declare that the research was conducted in the absence of any commercial or financial relationships that could be construed as a potential conflict of interest.

## Publisher’s Note

All claims expressed in this article are solely those of the authors and do not necessarily represent those of their affiliated organizations, or those of the publisher, the editors and the reviewers. Any product that may be evaluated in this article, or claim that may be made by its manufacturer, is not guaranteed or endorsed by the publisher.

## Supplementary Material

Supplementary Figure 1 Langendorff heart perfusion. Retrograde buffer perfusion of isolated wild type (WT) and complement component 3 knock out (C3KO) hearts exposed to ischemia-reperfusion. Hearts were exposed to 20 minutes stabilization, 35 minutes ischemia and 60 minutes of reperfusion and left ventricular (LV) function was measured. **(A)** heart rate, **(B)** rate pressure product (RPP), **(C)** coronary flow, and **(D)** LV developing pressure (LVdevP). Data sets are displayed as mean ± SEM.

Supplementary Figure 2 Extracellular C3a treatment of cardiomyocytes. **(A)** Activation of Akt (presented as p-Akt/total Akt) was measured in cardiomyocytes treated with extracellular C3a (10 and 100 µg/mL mouse recombinant C3a (R&D systems, Minneapolis, MN) or 10 and 100 µg/mL human C3a (Complement Technology, Inc., Tayler, TX)), n=4. Data set is displayed as scatterplots with median.

Supplementary Figure 3 Metabolites in mouse WT and C3KO hearts. Complement component 3 knock out (C3KO) and wild type (WT) hearts were analyzed with an untargeted metabolomic scan by Human Metabolome Technologies, n=6. Significant metabolites **(A–P)** are presented as relative area. Data sets are displayed as scatterplots with median.

Supplementary Figure 4 Lactate and succinate measurements in coronary perfusate. Isolated hearts from wild type (WT) and complement component 3 knock out (C3KO) were exposed to 20 minutes stabilization, 35 minutes ischemia, and 1 minute reperfusion. Coronary perfusates were collected at end of stabilization as a baseline. Subsequently at 0-30 seconds and 30-60 seconds during reperfusion. **(A)** Lactate and **(B)** succinate, n=4-8. Data sets are displayed as scatterplots with median.

Supplementary Figure 5 IL-6 release in coronary perfusate. Isolated hearts from wild type (WT) and complement component 3 knock out (C3KO) were exposed to 20 minutes stabilization, 35 minutes ischemia, and 60 minutes reperfusion. Coronary perfusates were collected and IL-6 release was investigated. Data set is displayed as mean ± SEM.
