# Supplementary figures and images for: Intracellular Complement Component 3 Attenuated Ischemia-Reperfusion Injury in the Isolated Buffer-Perfused Mouse Heart and Is Associated With Improved Metabolic Homeostasis
Source: Front Immunol. 2022 Apr 1;13:870811. doi: 10.3389/fimmu.2022.870811 (PMC9011808; doi:10.3389/fimmu.2022.870811)

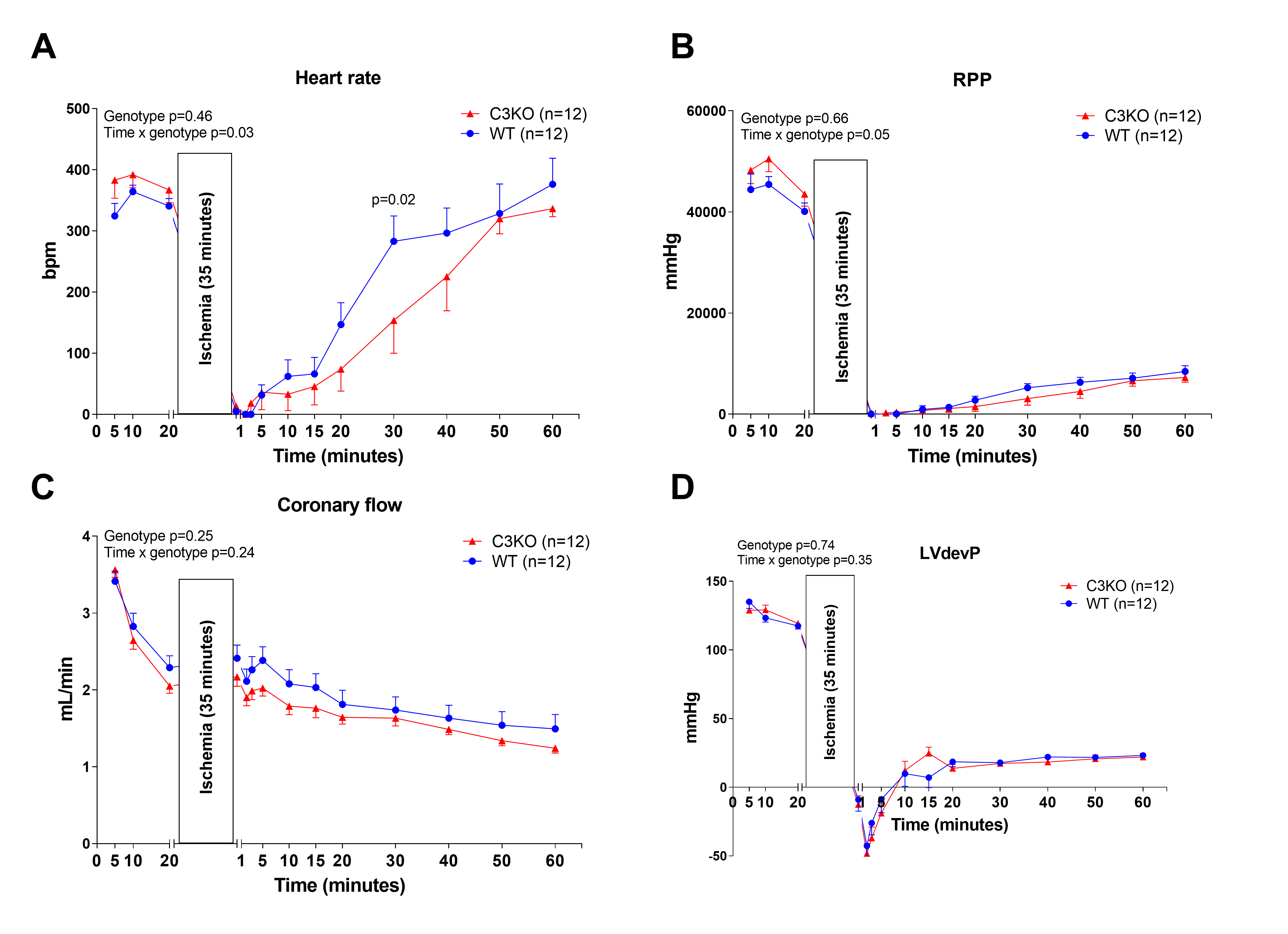

Supplement: Supplementary Figure 1 — Langendorff heart perfusion. Retrograde buffer perfusion of isolated wild type (WT) and complement component 3 knock out (C3KO) hearts exposed to ischemia-reperfusion. Hearts were exposed to 20 minutes stabilization, 35 minutes ischemia and 60 minutes of reperfusion and left ventricular (LV) function was measured. (A) heart rate, (B) rate pressure product (RPP), (C) coronary flow, and (D) LV developing pressure (LVdevP). Data sets are displayed as mean ± SEM. [file Image_1.tif]

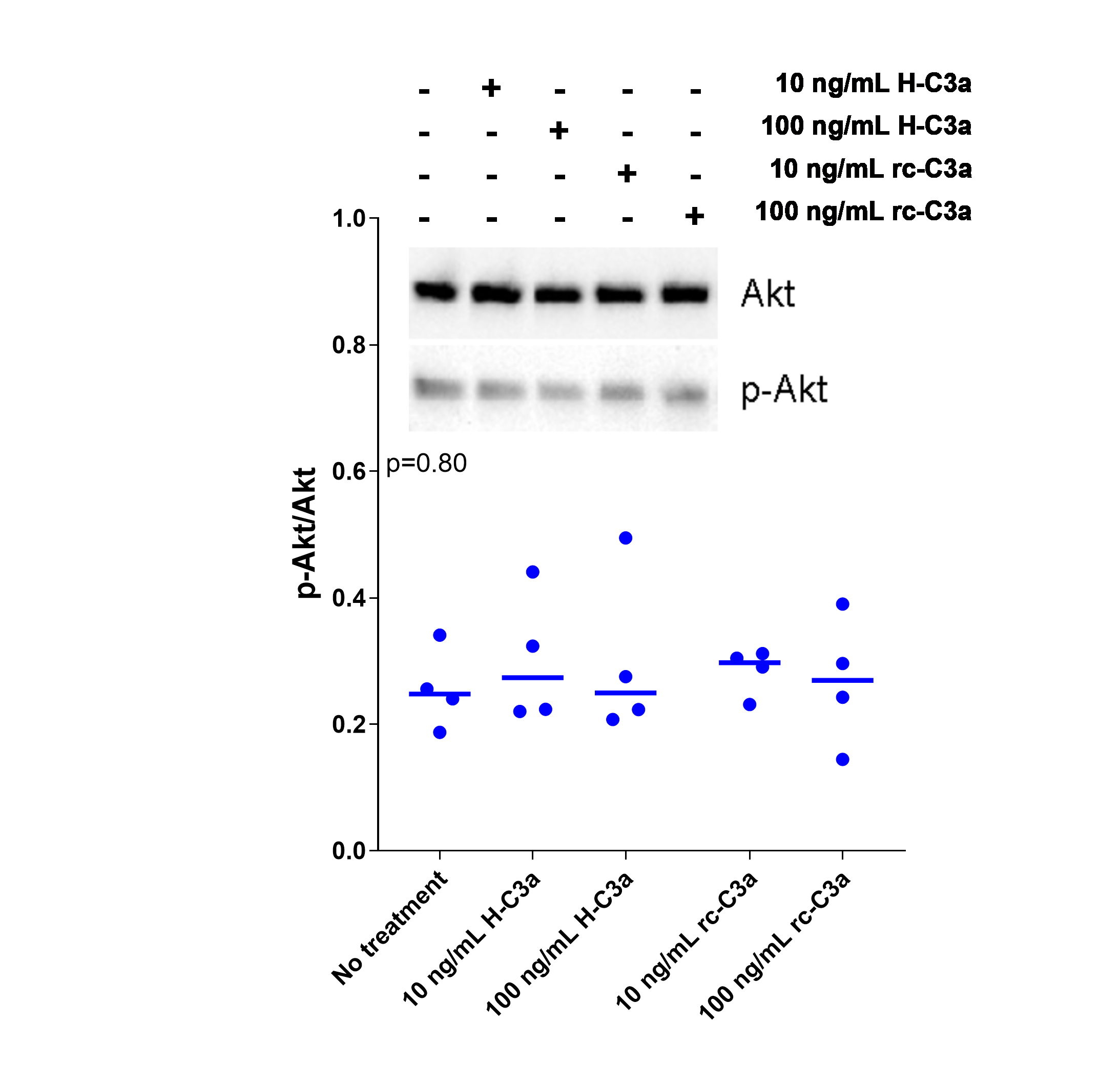

Supplement: Supplementary Figure 2 — Extracellular C3a treatment of cardiomyocytes. (A) Activation of Akt (presented as p-Akt/total Akt) was measured in cardiomyocytes treated with extracellular C3a (10 and 100 µg/mL mouse recombinant C3a (R&D systems, Minneapolis, MN) or 10 and 100 µg/mL human C3a (Complement Technology, Inc., Tayler, TX)), n=4. Data set is displayed as scatterplots with median. [file Image_2.tif]

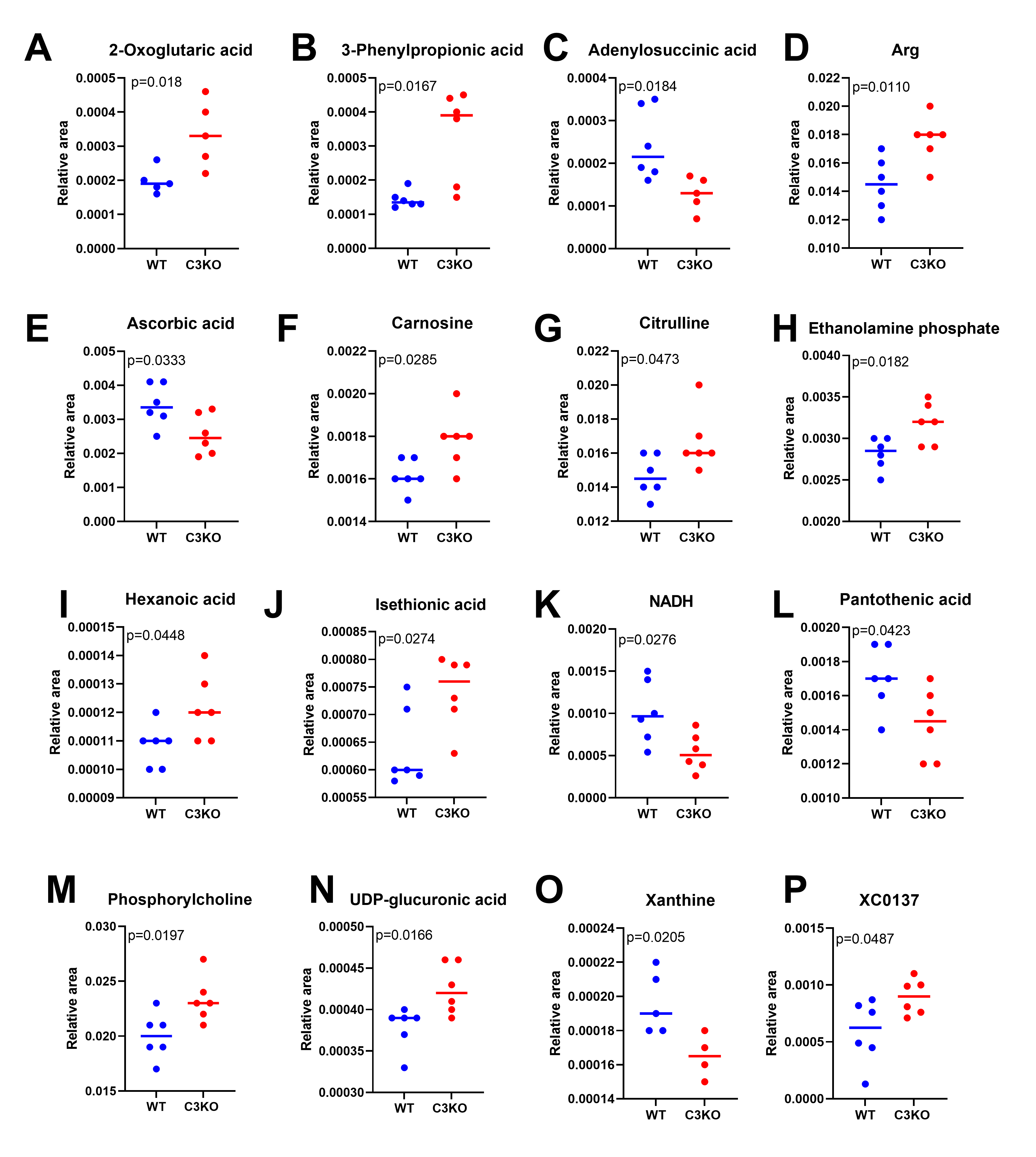

Supplement: Supplementary Figure 3 — Metabolites in mouse WT and C3KO hearts. Complement component 3 knock out (C3KO) and wild type (WT) hearts were analyzed with an untargeted metabolomic scan by Human Metabolome Technologies, n=6. Significant metabolites (A–P) are presented as relative area. Data sets are displayed as scatterplots with median. [file Image_3.tif]

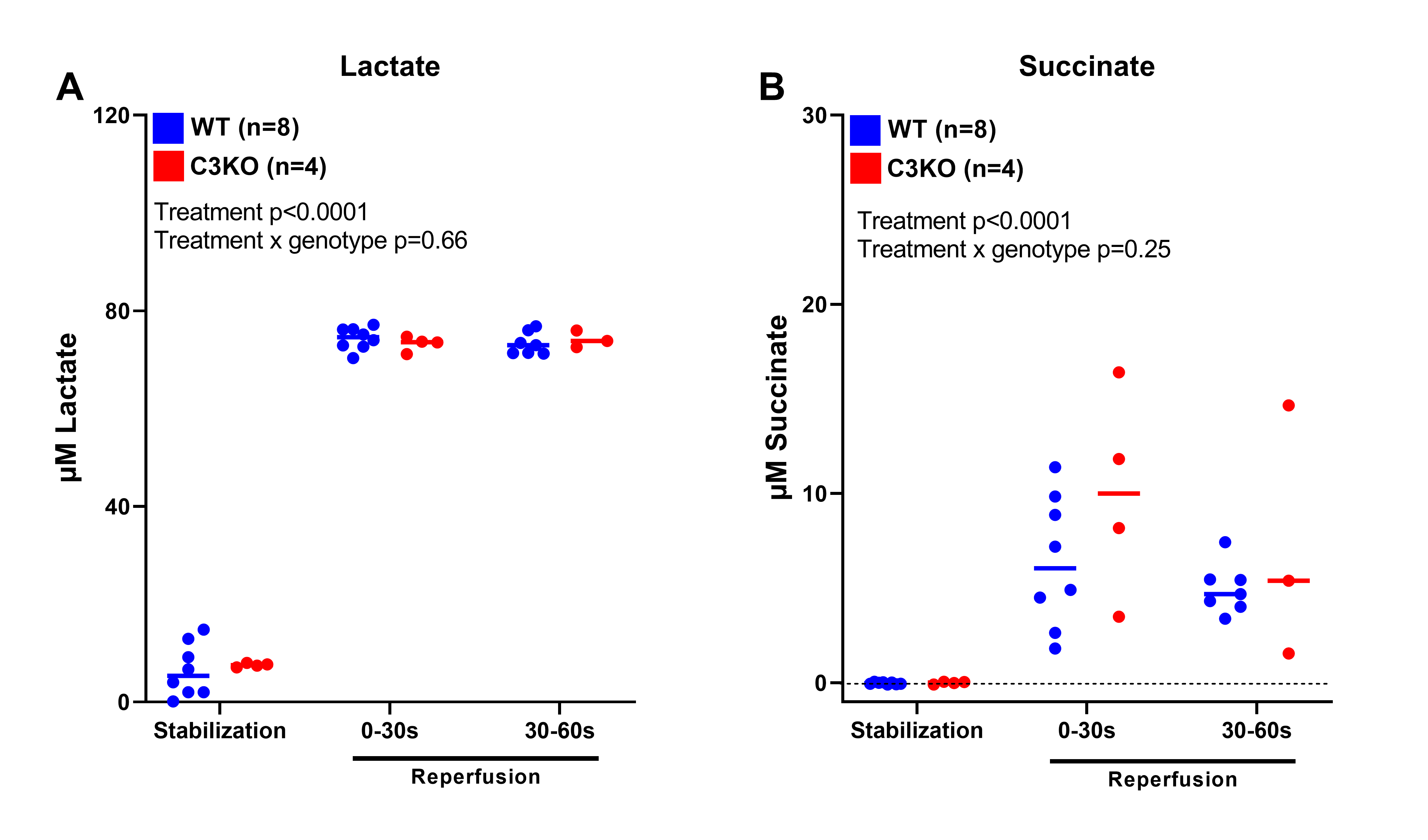

Supplement: Supplementary Figure 4 — Lactate and succinate measurements in coronary perfusate. Isolated hearts from wild type (WT) and complement component 3 knock out (C3KO) were exposed to 20 minutes stabilization, 35 minutes ischemia, and 1 minute reperfusion. Coronary perfusates were collected at end of stabilization as a baseline. Subsequently at 0-30 seconds and 30-60 seconds during reperfusion. (A) Lactate and (B) succinate, n=4-8. Data sets are displayed as scatterplots with median. [file Image_4.tif]

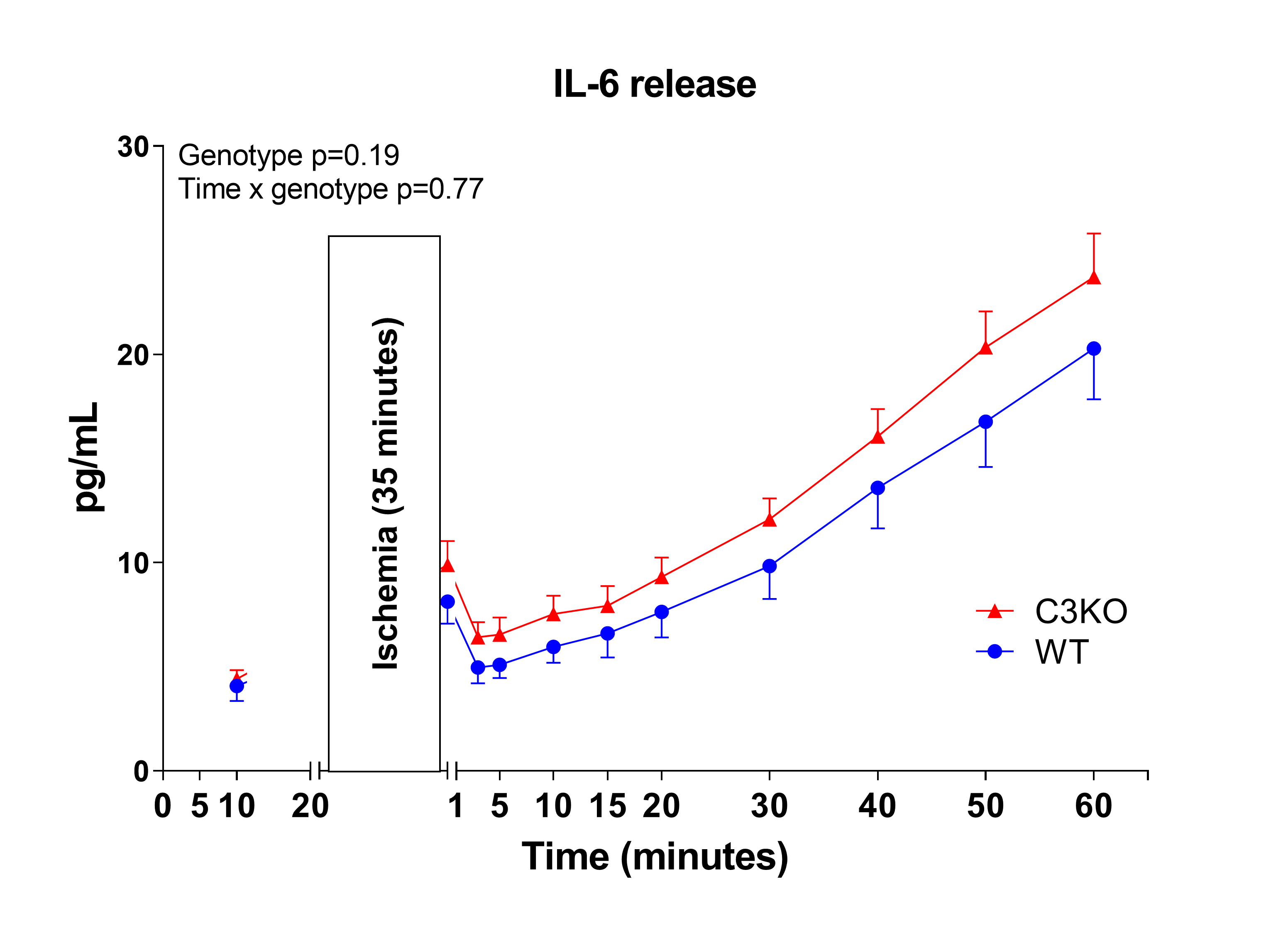

Supplement: Supplementary Figure 5 — IL-6 release in coronary perfusate. Isolated hearts from wild type (WT) and complement component 3 knock out (C3KO) were exposed to 20 minutes stabilization, 35 minutes ischemia, and 60 minutes reperfusion. Coronary perfusates were collected and IL-6 release was investigated. Data set is displayed as mean ± SEM. [file Image_5.tif]
